# Moderate maternal separation mitigates the altered synaptic transmission and neuronal activation in amygdala by chronic stress in adult mice

**DOI:** 10.1186/s13041-019-0534-4

**Published:** 2019-12-18

**Authors:** Xia Qin, Ye He, Na Wang, Jia-Xin Zou, Yong-Mei Zhang, Jun-Li Cao, Bing-Xing Pan, Wen-Hua Zhang

**Affiliations:** 1 College of Life Science, Nanchang University, Nanchang, 330031 China; 2 Jiangsu Province Key Laboratory of Anesthesiology, Xuzhou Medical University, Xuzhou, 221004 Jiangsu China; 3 Laboratory of Fear and Anxiety Disorders, Institute of Life Science, Nanchang University, Nanchang, 330031 China; 4 Department of Pharmacology, Nanchang University, Nanchang, 330031 China; 5 Department of Physiology, Mudanjiang Medical University, Mudanjiang, 157011 China

**Keywords:** Moderate maternal separation, Stress inoculation, Amygdala, Anxiety, Glutamatergic transmission, Excitability

## Abstract

Exposure to moderate level of stress during the perinatal period helps the organisms to cope well with stressful events in their later life, an effect known as stress inoculation. Amygdala is one of the kernel brain regions mediating stress-coping in the brain. However, little is known about whether early life stress may affect amygdala to have its inoculative effect. Here, we observed that moderate maternal separation (MS) from postnatal day 3 to day 21 (D3–21, 1 h per day) significantly alleviated the increased anxiety-like behavior induced by chronic social defeat stress (CSDS) in adulthood, suggesting an obvious inoculative effect of moderate MS. Further studies revealed that MS prevented CSDS-evoked augmentation of glutamatergic transmission onto principal neurons (PNs) in the basolateral amygdala (BLA) by inhibiting presynaptic glutamate release. By contrast, it did not affect GABAergic transmission in BLA PNs, as indicated by unaltered frequency and amplitude of miniature inhibitory postsynaptic currents (mIPSCs). Moreover, the CSDS-induced increase of neuronal excitability was also mitigated by MS in BLA PNs. In conclusion, our results suggest that MS may have its inoculative effect through alleviating the influences of later life stress on the glutamatergic transmission and neuronal activity in amygdala neurons.

## Introduction

The effects of stress on the brain and behavior often vary in a nonlinear manner, with the severity of stress roughly corresponding to an inverted U-shaped reaction norm. Specifically, mild-to-moderate stress has beneficial and salutary effects, while severe and/or prolonged stress has deleterious effects [1, 2]. For example, pre-weaning repeated, long-term maternal separation (i.e., 3–24 h, a severe stress) has been found to induce maladaptive behavioral changes such as anxiety [3], and deficits in associative learning. However, early postnatal rats exposed to 14 days of 15-min maternal separation or 21 days of 1 h maternal separation [4] (a moderate stressor) subsequently show attenuated stress response. In the past decades, the bulk of neurobiological research about stress focuses on its deleterious effects, demonstrating that stress is one of the significant risk factors for various neuropsychiatric conditions [5]. Yet, exposure to mild to moderate levels of stress in early life can promote resilience to the succeeding stress later in life, which has been regarded as stress inoculation [6].

Stress inoculation makes subsequent coping efforts more efficient and promotes adaptation later in life in humans [7–9], primates [10, 11] and rodents [5, 12]. Research on the brain mechanisms of stress inoculation revealed that multiple brain regions including prefrontal cortex [13, 14], striatum [10] and hippocampus [15] are engaged in this process. For instance, in the anterior cingulate cortex (ACC), stress inoculation by intermittent social separation reduces the hypothalamic-pituitary-adrenal (HPA) axis stress hormone response and increases glucocorticoid receptor (NR3C1) gene expression [14]. In the ventromedial prefrontal cortex, stress inoculation increases myelination and cortical volumes [13]. And in the ventral striatum, stress inoculation by brief intermittent maternal separation increases dopamine D2 and/or D3 receptors availability [10]. In the hippocampus, stress inoculation-like training sessions increase adult monkey hippocampal neurogenesis and alter the expression of genes involved in cell proliferation and survival [15]. However, little is known about amygdala engaged in the inoculative effect by early life stress.

Amygdala is a diamond-shaped brain structure located deep inside the temporal lobe and has an essential role in emotional processing, such as fear and anxiety. The amygdala is composed of more than ten interconnected nuclei including basolateral (BLA) and central amygdala (CeA). Unlike the central amygdala with most neurons GABAergic, basolateral amygdala (BLA) consists of extensively excitatory glutamatergic neurons (80–90%) and inhibitory GABAergic neurons (10–20%) [16, 17]. The abundant inhibitory connections made by the local interneurons endow the amygdala with a highly inhibitory tone, which is crucial for preventing inappropriate expression of emotions. However, upon severe and/or prolonged stress exposure, the amygdala becomes disinhibited and manifests hyperactivity which is designated as a pivotal neurobiological basis underlying multiple mental diseases such as anxiety disorders and depression [18].

Here, we aimed to examine whether the inoculative effect of early moderate MS is mediated through preventing overactivation of the amygdala by later stress. Our results showed that postnatal MS alleviated anxiety-like behavior caused by later-life CSDS. Moreover, it mitigated CSDS-induced enhancement of excitatory synaptic transmission by reducing presynaptic glutamate release, prevented subsequent stress-induced increase of intrinsic excitability.

## Materials and methods

### Animals

Male C57BL/6 J mice were used and housed in groups of 3–5 per cage in a temperature- and humidity-controlled animal facility with ad libitum access to food and water under a 12-h light/dark cycle. Females were housed individually once they became pregnant. All experimental procedures were approved by the Institutional Animal Care and Use Committee of Nanchang University.

### Moderate maternal separation (MS)

The cages were checked every day for births and the day of birth was noted as postnatal day 0 (D0). 8–10 pups per litter were used regardless of the sex. Dams with litters were left undisturbed until PND 3. In the MS group, pups were removed from their dams and home cages in a separate room for 1 h per day from postnatal day 3 (D3) to D21. The room was maintained at 28 ± 2 °C. Afterwards the pups were reunited with their dams in the home cage. After weaning at D21, littermates were housed in the same-sex groups and only males were used for the following experiments. Littermates were randomly divided into 4 groups: Control, CSDS, MS and MS + CSDS.

### Chronic social defeat stress (CSDS)

Social defeat stress paradigm was performed as previously described [19]. From D50 to D60, each experimental male C57BL/6 J mouse (intruder) was moved into the home cage of a novel aggressive CD1 mouse (resident) every day for 5 min per day. The intruder was always physically defeated by the CD1 mouse. After 5 min of physical interaction, a perforated Acrylicglass partition was used to divide the resident home cage into two parts and the residents and intruders were maintained only in sensory contact for 24 h. After 10 days of social defeat stress, animals were tested. The control animals were housed in pairs in a cage evenly separated by a perforated Acrylicglass partition. They were never in physical or sensory contact with CD1 mice. After stress, each mouse was used for either behavioral tests or electrophysiology experiments.

### Open field test (OFT)

Each tested mouse was placed in the central zone of an open-field (50 × 50 cm^2^) chamber. The animal’s location, path and the time spent in the center square were monitored for 10 min by an overhead tracking system (Med Associates Inc., Farifax, VT). The locomotor activity of mice was evaluated as the total distance they travelled. The anxiety-like behavior was evaluated by the time they spent in the central zone (25 × 25 cm^2^).

### Elevated plus maze (EPM) test

The elevated plus maze apparatus is comprised of two open arms (30 × 6 cm^2^), two closed arms (30 × 6 cm^2^) and a connecting central platform (6 × 6 cm^2^) elevated 74 cm above the floor. Mice were placed in the center platform facing one of the two open arms and allowed to explore the apparatus for 10 min. Their anxiety-like behavior was assessed by the number of entries and the time spent in the open arms with video tracking software (DOC-086; Med Associates, Inc).

### Ex vivo slice electrophysiology

Amygdala slices were prepared as previously described [20]. Briefly, the mice were anesthetized with ether and decapitated, then brains were quickly removed from the skull and chilled in ice-cold artificial cerebrospinal fluid (ACSF) containing (in mM) 124 NaCl, 2.5 KCl, 2 MgSO_4_, 2.5 CaCl_2_, 1.25 NaH_2_PO_4_, 22 NaHCO_3_, and 10 glucose, gassed with 95% O_2_ and 5% CO_2_. 320 μm thick coronal brain slices containing the amygdala were cut using the VT1000S Vibratome (Leica Microsystems). The slices were recovered in ACSF for 30 min at 34 °C and removed to the incubator at room temperature for at least 1 h before recording. Whole-cell patch clamp recordings were performed using an infrared differential interference contrast microscope (BX51WI, Olympus, Tokyo, Japan) with a water immersion 40 × objective (40 × /N.A. 0.8, LUMPlanFL, Olympus) equipped with 2 automatic manipulators (Sutter Instrument Co., Novato, CA) and a highly sensitive CCD camera (IR-1000E, DAGE-MTI, Michigan, IN, USA). A single slice was transferred to the recording chamber and continuously perfused with the ACSF at a flow rate of ∼2 ml/min. Recording electrodes were made from filamented borosilicate glass capillary tubes (inner diameter, 0.84 μm) by using a horizontal pipette puller (P-97; Sutter Instrument). Principal neurons (PNs) were selected based on the cell size and membrane capacitance as previously described [21].

To record mEPSCs, the membrane potentials were held at − 70 mV and 1 μM tetrodotoxin (TTX) and 100 μM picrotoxin (PTX) were added in the bath solution. The patch electrodes were filled with K-gluconate pipette solution containing (in mM), 130 K-gluconate, 5 NaCl, 1 MgCl_2_, 10 HEPES, 0.2 EGTA, 2 MgATP, and 0.1 NaGTP. The pH was adjusted to 7.3–7.4 with KOH and osmolarity to 285 mOsm with sucrose.

To evoke action potentials, cells were recorded at current clamp mode and the depolarizing current pulses with the ramped strength (0–250 pA, 1500 ms) were delivered. The membrane potentials were held at − 70 mV with or without 20 μM CNQX, 25 μM APV and 100 μM PTX in the bath solution. The patch electrodes were filled with aforementioned 130 mM K-gluconate pipette solution. Spike properties were calculated based on the first spike elicited by the ramp protocol. Action potential (AP) threshold was identified at the point where the action potential was initiated and showed a > 10-fold change in the rising rate. AP amplitude was calculated as the maximum voltage during the spike minus the spike threshold voltage. The rheobase was measured as the current at the first AP threshold. The fast after-hyperpolarization potential (fAHP) was measured as the difference between the peak following the spike and the threshold potential.

To evoke EPSC, a stimulating electrode was placed locally in the BLA and about 100 μm away from the recording electrode. Paired stimuli were delivered every 30 s with an inter-stimulus interval of 50 ms (6 consecutive sweeps). 100 μM PTX and 5 μM CGP52432 were added to the bath solution to block GABA_A_R current and GABA_B_R current. The membrane potentials were held at − 70 mV and the patch electrodes were filled with Cs-methanesulfonate pipette solution containing (in mM) 125 Cs-methanesulfonate, 5 CsCl, 5 NaCl, 1 MgCl_2_, 10 HEPES, 0.2 EGTA, 2 MgATP, and 0.1 NaGTP. The stimulus intensity was determined according to the first evoked EPSC amplitude that was maintained within 50–200 pA. Paired pulse ratio per cell was obtained by averaging 6 consecutive sweeps.

To record miniature IPSC (mIPSCs), the membrane potentials were held at − 70 mV and 20 μM CNQX, 20 μM APV and 1 μM TTX were added to the bath solution. The patch electrodes were filled with CsCl pipette solution containing (in mM) 100 CsCl, 30 Cs-methanesulfonate, 5 NaCl, 1 MgCl_2_, 10 HEPES, 0.2 EGTA, 2 MgATP, and 0.1 NaGTP.

The frequency and amplitude of mEPSC or mIPSCs were determined from all detected events in each cell in the middle 1-min period during 3-min consecutive recording. Series resistance (Rs) was in the range of 10-20 MΩ and monitored throughout the experiments. If Rs changed more than 20% during recording, the data were not included in analysis. Data were sampled at 10 K Hz and filtered at 2 K Hz using the patch-clamp amplifier (EPC-10 USB, HEKA Instrument Inc., Germany) circuitry. The mEPSCs and mIPSCs were analyzed offline using Minianalysis (Synaptosoft). The eEPSCs and action potentials (APs) were analyzed with Origin8.5 (Origin Lab, Northampton, MA, USA).

### Statistics

Data were expressed as mean ± SEM. Statistical analyses were performed using two-way analysis of variance (ANOVA) followed by post hoc comparison with Bonferroni t test. Differences between means were considered significant if *p* < 0.05. Statistical analyses were performed using Graphpad Prism Version 6 (GraphPad Software, La Jolla, CA).

## Results

### MS ameliorates CSDS-induced anxiety-like behavior in adult mice

**Fig. 1:**
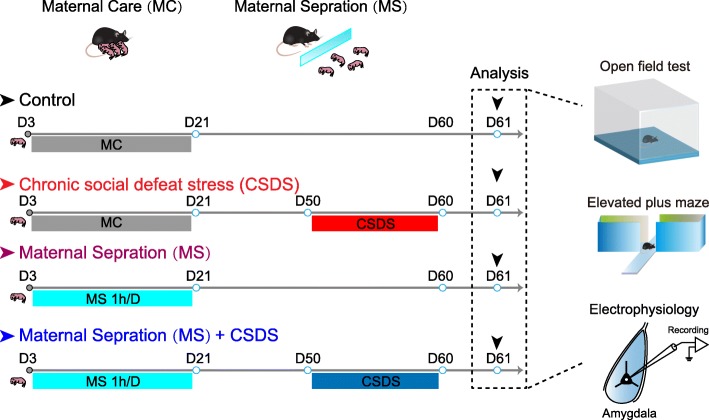
Schematic of experimental designs

**Fig. 2:**
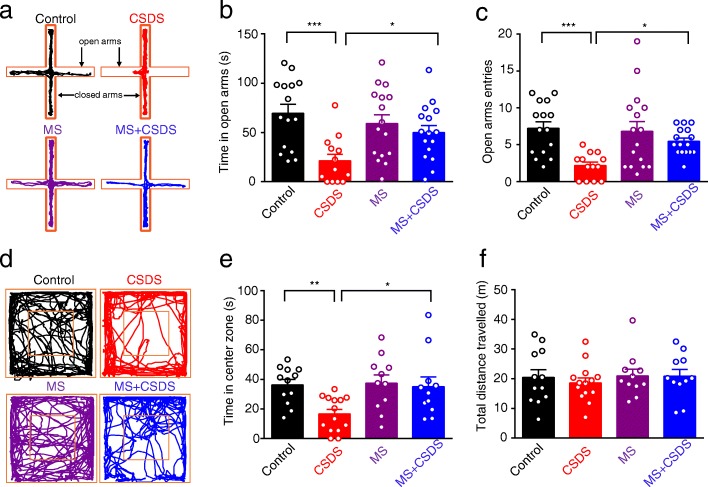
Alleviation of CSDS-induced anxiety-like behavior by MS in adult mice. **a** Activity tracks from representative mice during performance on the elevated plus-maze. **b** Comparison of the time spent in open arms in the elevated plus maze (EPM). *p < 0.05, ***p < 0.001. **c** Comparison of entrances in open arms in the elevated plus maze (EPM). *p < 0.05, ***p < 0.001. **d** Average time mice spent in the center square in the open field test (OFT). **e** Comparison of the time spent in center square in the open field test (OFT). *p < 0.05, **p < 0.01. **f** Comparison of total distance travelled in the open field test (OFT). All data are presented as the mean ± SEM

To assess whether early mild MS used in the present study had inoculative effect for the late-arriving stress in adult mice, we separated the mice into four groups (Fig. 1). The anxiety-like behavior of each group of mice was evaluated by OFT and EPM on D61 (Fig. 1). Two-way ANOVA revealed significant effects of CSDS and MS × CSDS interaction on open-arm time (main effect of CSDS: *F*_1,56_ = 12.39, *p* = 0.0009; main effect of MS: *F*_1,56_ = 1.258, *p* = 0.2668; interaction: *F*_1,56_ = 5.698, *p* = 0.0204; Control: *n* = 15 mice, CSDS: *n* = 13 mice, MS and MS + CSDS: *n* = 16 mice, Fig. 2a, b) and open-arm entries in EPM (main effect of CSDS: *F*_1,56_ = 12.9, *p* = 0.0007; main effect of MS: *F*_1,56_ = 2.624, *p* = 0.1109; interaction: *F*_1,56_ = 4.216, *p* = 0.0447, Fig. 2a, c). In addition, OFT results showed that significant main effects of CSDS and MS on time spent in the center square were observed (main effect of CSDS: *F*_1,44_ = 5.43, *p* = 0.0244; main effect of MS: *F*_1,44_ = 4.283, *p* = 0.0444; interaction: *F*_1,44_ = 3.372, *p* = 0.0731; Control: *n* = 12 mice, CSDS: *n* = 14 mice, MS, MS + CSDS: *n* = 11 mice, Fig. 2d, e), which signaled the anxiety-like behavior. Relative to the control mice, CSDS mice exhibited elevated anxiety-like behavior, as manifested by decreased open-arm time (*p* = 0.0003, Fig. 2a, b) and entries in EPM (*p* = 0.0006, Fig. 2a, c) as well as less time spent in the center square in OFT (*p* = 0.0073, Fig. 2d, e). MS per se had little effect on the anxiety-like behavior of mice, as indicated by the unaltered behavioral parameters between the MS and control mice. However, it effectively mitigated the increased anxiety-like behavior induced by CSDS in adult mice (MS + CSDS vs. CSDS), as indicated by more open arm time (*p* = 0.0362, Fig. 2a, b) and more entries in EPM (*p* = 0.0271, Fig. 2a, c) and more time in the center square in OFT (*p* = 0.0147, Fig. 2d, e) in mice experiencing both MS and CSDS relative to those having CSDS only. No main effects of MS and CSDS were observed in the total distance mice travelled in OFT (main effect of CSDS: *F*_1,44_ = 0.21, *p* = 0.6490; main effect of MS: *F*_1,44_ = 0.4039, *p* = 0.5284; interaction: *F*_1,44_ = 0.1555, *p* = 0.6952; Fig. 2d, f). Furthermore, neither MS nor CSDS had significant main effect on the total distance travelled in EPM (Two-way ANOVA; main effect of CSDS: *F*_1,56_ = 0.001618, *p* = 0.9681; main effect of MS: *F*_1,56_ = 0.2617, *p* = 0.6109; interaction: *F*_1,56_ = 0.02581, *p* = 0.8729; Additional file 1: Figure S1a), closed arm entries in EPM (Two-way ANOVA; main effect of CSDS: *F*_1,56_ = 0.3300, *p* = 0.5679; main effect of MS: *F*_1,56_ = 0.8567, *p* = 0.3586; interaction: *F*_1,56_ = 0.1324, *p* = 0.7174; Additional file 1: Figure S1b) and time course of locomotion in OFT (Two-way ANOVA with repeated measures, main effect of time: *F*_9,396_ = 11.19, *p* < 0.0001; main effect of group: *F*_3,44_ = 0.3072, *p* = 0.8201; interaction: *F*_27,396_ = 1.090, *p* = 0.3478; Additional file 1: Figure S1c). Taken together, these data indicate that the early MS effectively antagonizes the adverse influence of later stress and thus has stress-inoculative effect.

### MS alleviates CSDS-evoked augmentation of glutamatergic transmission in BLA PNs

**Fig. 3:**
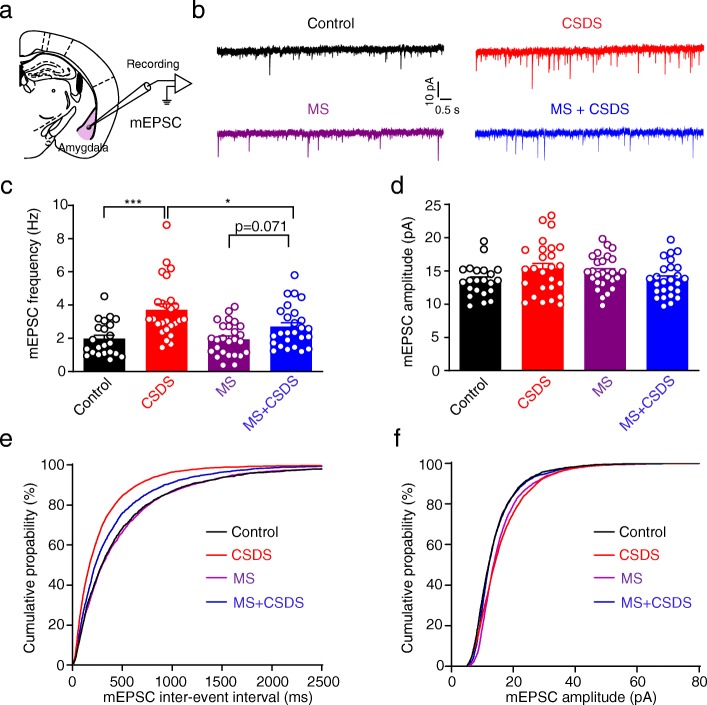
Amelioration of CSDS-induced augmentation of mEPSC frequency by MS in BLA PNs. **a** Schematic diagram showing the recordings made from BLA principal neurons. **b** Representative traces showing mEPSCs in BLA PNs. **c** Average mEPSC frequency in BLA PNs. *p < 0.05, ***p < 0.001. **d** Average mEPSC amplitude in BLA PNs. **e** Cumulative probability of the inter-event interval of mEPSCs in BLA. Kolmogorov-Smirnov test vs. Control: CSDS, *p* < 0.001; Kolmogorov-Smirnov test vs. CSDS: MS + CSDS, *p* < 0.001; Kolmogorov-Smirnov test vs. Control: MS, *p* > 0.9999; Kolmogorov-Smirnov test vs. MS: MS + CSDS, *p* = 0.0011. **f** Cumulative probability of mEPSC amplitude in BLA. Kolmogorov-Smirnov test vs. Control: CSDS, *p* = 0.0078; Kolmogorov-Smirnov test vs. CSDS: MS + CSDS, *p* = 0.3331; Kolmogorov-Smirnov test vs. Control: MS, *p* = 0.5115; Kolmogorov-Smirnov test vs. MS: MS + CSDS, *p* = 0.9997. All data are presented as the mean ± SEM

Amygdala is a key target of stress hormone and mediator of stress influence on brain and behavior. We next investigated the possible influence of MS on principal neurons (PNs) in the BLA from both control and stressed mice. We first tested the effect of MS on the excitatory synaptic transmission onto BLA PNs (Fig. 3a). Significant main effects of CSDS and MS were observed in the frequency (main effect of CSDS: *F*_1,96_ = 23.61, *p* < 0.001; main effect of MS: *F*_1,96_ = 4.167, *p* = 0.044; interaction: *F*_1,96_ = 3.644, *p* = 0.0593; Control: *n* = 22 neurons / 4 mice, CSDS, MS and MS + CSDS: *n* = 26 neurons / 4 mice; Fig. 3b, c) but not amplitude of mEPSCs (main effect of CSDS: *F*_1,96_ = 0.3580, *p* = 0.5511; main effect of MS: *F*_1,96_ = 0.09694, *p* = 0.7562; interaction: *F*_1,96_ = 6.085, *p* = 0.0154; Fig. 3b, d, f). Post hoc analysis demonstrated that CSDS markedly increased the mEPSC frequency (*p* < 0.001, CSDS vs. Control), and such increase could be weakened by MS (*p* = 0.0105, MS + CSDS vs. CSDS) (Fig. 3b, c) although a higher mEPSC frequency was still observed in MS + CSDS mice relative to the MS ones (*p* = 0.071). In line with this, CSDS left-shifted the cumulative probability curve of inter-event interval of mEPSCs and MS significantly, although not completely, reversed the shift by CSDS (Fig. 3e). MS itself had little influence on the mean frequency of mEPSCs (*p* > 0.9999 vs. Control). Altogether, these data reveal that CSDS conspicuously enhanced glutamatergic transmission onto BLA PNs, an effect which could be readily alleviated by moderate MS in early life, suggesting that MS has an inoculative effect against the regulation of CSDS on BLA PNs in adult mice.

**Fig. 4:**
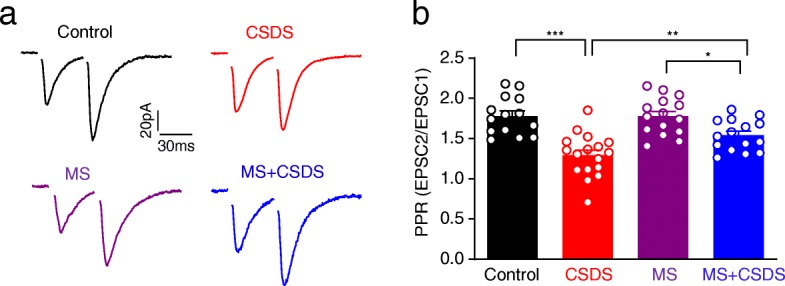
Mitigation of CSDS-induced augmentation of presynaptic glutamate release by MS with 50 ms interval in BLA PNs. **a** Representative traces showing electrically evoked EPSCs in BLA PNs with 2 stimuli separated by 50 ms. **b** Average paired pulse ratio (PPR) in BLA PNs with 2 stimuli separated by 50 ms. *p < 0.05, **p < 0.01, ***p < 0.001. All data are presented as the mean ± SEM

The influence of MS and CSDS on the frequency but not amplitude of mEPSCs indicate that they may preferentially act to regulate the presynaptic glutamate releases in afferents to BLA PNs. To further confirm this, we recorded the evoked EPSCs in BLA PNs upon two consecutive electrical stimuli (separated by 50 ms) delivered through a stimulation electrode placed close to the recorded cells. The ratio of the amplitude of the 2nd EPSC over that of the 1st one was defined as the paired pulse ratio (PPR). Changes in PPR are inversely related to the transmitter release such that a reduction of PPR is associated with an increased probability of transmitter release [22]. As shown in Fig. 4, two-way ANOVA revealed significant main effect of CSDS and MS on PPR (main effect of CSDS: *F*_1,57_ = 36.19, *p* < 0.001; main effect of MS: *F*_1,57_ = 4.162, *p* = 0.046; interaction: *F*_1,57_ = 4.33, *p* = 0.0419; Control: *n* = 14 neurons / 4 mice, CSDS: *n* = 17 neurons / 4 mice, MS and MS + CSDS: *n* = 15 neurons / 4 mice). Post hoc demonstrated that CSDS significantly reduced the PPR (*p* < 0.001 vs. Control), which was partially prevented by MS (*p* = 0.0083, MS + CSDS vs. CSDS) in BLA PNs. No effect of MS on the PPR was observed (*p* > 0.9999, MS vs. Control). These data demonstrate that MS mitigates later life stress-induced augmentation of presynaptic glutamate release.

### MS and CSDS did not affect GABAergic transmission onto BLA PNs

**Fig. 5:**
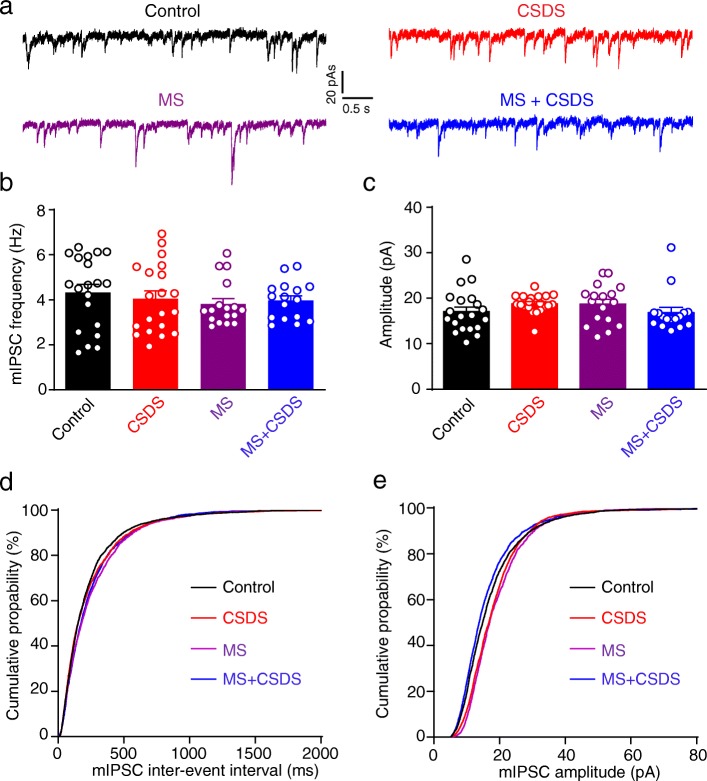
MS and CSDS did not affect mIPSC frequency or amplitude in BLA PNs. **a** Representative traces showing mIPSCs in BLA PNs. **b** Average mIPSC frequency in BLA PNs. **c** Average mIPSC amplitude in BLA PNs. **d** Cumulative probability of the inter-event interval of mIPSCs in BLA. Kolmogorov-Smirnov test vs. Control: CSDS, *p* = 0.0002; Kolmogorov-Smirnov test vs. CSDS: MS + CSDS, *p* = 0.9762; Kolmogorov-Smirnov test vs. Control: MS, *p* < 0.0001; Kolmogorov-Smirnov test vs. MS: MS + CSDS, *p* < 0.0001. **e** Cumulative probability of mIPSC amplitude in BLA. Kolmogorov-Smirnov test vs. Control: CSDS, *p* = 0.1568; Kolmogorov-Smirnov test vs. CSDS: MS + CSDS, *p* = 0.6631; Kolmogorov-Smirnov test vs. Control: MS, *p* = 0.0317; Kolmogorov-Smirnov test vs. MS: MS + CSDS, *p* = 0.4189. All data are presented as the mean ± SEM

We next asked whether MS also impacted the inhibitory synaptic transmission onto BLA PNs. As shown in Fig. 5, neither MS nor CSDS had any obvious effect on the mIPSC frequency (main effect of CSDS: *F*_1,69_ = 0.04419, *p* = 0.8341; main effect of MS: *F*_1,69_ = 0.9574, *p* = 0.3313; Interaction: *F*_1,69_ = 0.4748, *p* = 0.4931; Control, CSDS: *n* = 20 neurons / 4 mice, MS: *n* = 17 neurons / 4 mice, MS + CSDS: *n* = 16 neurons / 4 mice; Fig. 5a, b) or amplitude (main effect of CSDS: *F*_1,69_ = 0.0056, *p* = 0.9405; main effect of MS: *F*_1,69_ = 0.0231, *p* = 0.8797; interaction: *F*_1,69_ = 3.679, *p* = 0.0592; Fig. 5a, c). Similarly, the cumulative probability curve of mIPSCs frequency and amplitude remained unaltered among the four groups (Fig. 5d, e). These data indicate inability of both MS and CSDS to impact the inhibitory transmission in life.

### MS attenuates CSDS-induced enhancement of intrinsic excitability in BLA PNs

**Fig. 6:**
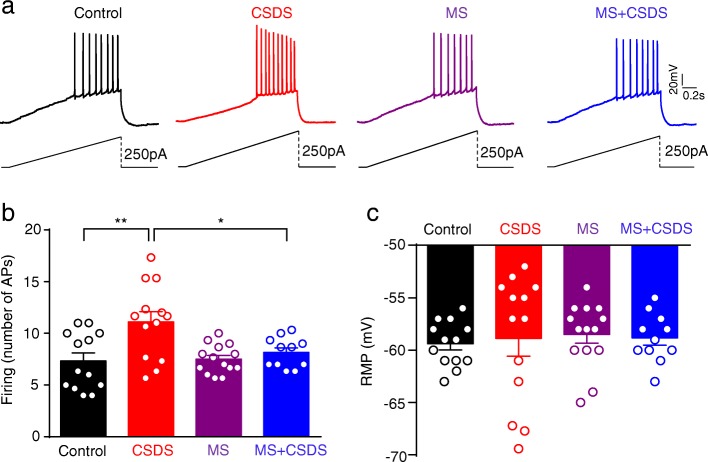
Prevention of CSDS-induced enhancement of excitability by MS in BLA PNs. **a** Representative spiking traces showing the firing of BLA PNs upon the injection of depolarizing current with the ramped strength (0 - 250pA, 1500 ms). **b** Comparison of the firing frequency (numbers of APs) (action potential, AP). *p < 0.05, **p < 0.01. **c** Summary plots of the changes of RMP in BLA PNs. All data are presented as the mean ± SEM

Regulation of the intrinsic excitability represents an important aspect of neuronal adaptation to the changing environment [23]. Chronic stress exposure has been shown to persistently increase the intrinsic excitability of BLA PNs [23]. We next explored the possible influence of MS on the altered excitability of BLA PNs by CSDS in adult mice. The excitability was measured by neuronal firing upon injection of a ramped depolarization currents. As shown in Fig. 6a, b, two-way ANOVA revealed significant main effect of both MS and CSDS on neuronal firing (main effect of CSDS: *F*_1,47_ = 9.900, *p* = 0.0029; main effect of MS: *F*_1,47_ = 3.927, *p* = 0.0534; interaction: *F*_1,47_ = 4.924, *p* = 0.0314; Control, CSDS: *n* = 13 neurons / 4 mice, MS: *n* = 14 neurons / 4 mice, MS + CSDS: *n* = 11 neurons / 4 mice). Post hoc analysis demonstrated that CSDS significantly increased the number of neuronal firing (*p* = 0.0007, CSDS vs. Control), which is consistent with previous findings [24, 25]. MS itself failed to affect the firing of BLA PNs (*p* > 0.9999 vs Control), but clearly alleviated CSDS-induced augmentation of excitability (*p* = 0.0119, MS + CSDS vs. CSDS).

Action potential generation in response to a ramped depolarization currents was again tested in BLA PNs from Control, CSDS, MS and MS + CSDS mice. However, to block any influence of ongoing synaptic activity, 20 μM CNQX, 25 μM APV and 100 μM picrotoxin were added to the superfusate to block glutamate and GABA_A_ receptors, respectively. Similarly, two-way ANOVA revealed significant main effect of both MS and CSDS on neuronal firing (main effect of CSDS: *F*_1,53_ = 6.040, *p* = 0.0173; main effect of MS: *F*_1,53_ = 4.143, *p* = 0.0468; interaction: *F*_1,53_ = 2.194, *p* = 0.1445; Control, CSDS and MS: *n* = 14 neurons / 3 mice, MS + CSDS: *n* = 15 neurons / 3 mice; Additional file 2: Figure S2a, b). Post hoc analysis revealed that CSDS significantly increased the number of neuronal firing (*p* = 0.0158, CSDS vs. Control), which was alleviated by MS (*p* = 0.0305, MS + CSDS vs. CSDS).

Besides, neither CSDS nor MS had significant main effect on resting membrane potential (RMP) (main effect of CSDS: *F*_1,47_ = 0.008525, *p* = 0.9268; main effect of MS: *F*_1,47_ = 0.1919, *p* = 0.6633; interaction: *F*_1,47_ = 0.1523, *p* = 0.6981; Control, CSDS: *n* = 13 neurons / 4 mice, MS: *n* = 14 neurons / 4 mice, MS + CSDS: *n* = 11 neurons / 4 mice; Fig. 6c). Two-way ANOVA revealed no significant main effect of both MS and CSDS on the threshold of action potential (AP) (main effect of CSDS: *F*_1,47_ = 0.05751, *p* = 0.8115; main effect of MS: *F*_1,47_ = 0.1969, *p* = 0.6593; interaction: *F*_1,47_ = 0.1523, *p* = 0.6981; Control, CSDS: *n* = 13 neurons / 4 mice, MS: *n* = 14 neurons / 4 mice, MS + CSDS: *n* = 11 neurons / 4 mice; Additional file 3: Figure S3a), rheobase (main effect of CSDS: *F*_1,47_ = 0.07753, *p* = 0.7819; main effect of MS: *F*_1,47_ = 0.02235, *p* = 0.8818; interaction: *F*_1,47_ = 1.153, *p* = 0.2884; Additional file 3: Figure S3b), the amplitude of AP (main effect of CSDS: *F*_1,47_ = 0.02528, *p* = 0.8743; main effect of MS: *F*_1,47_ = 0.3675, *p* = 0.5473; interaction: *F*_1,47_ = 3.528, *p* = 0.0665; Additional file 3: Figure S3c) and fAHP (main effect of CSDS: *F*_1,47_ = 0.008232, *p* = 0.9281; main effect of MS: *F*_1,47_ = 0.8685, *p* = 0.3561; interaction: *F*_1,47_ = 0.8574, *p* = 0.3592; Additional file 3: Figure S3d). Taken together, these data indicate that MS mitigates later life stress-induced augmentation of intrinsic excitability.

## Discussion

In the present study, we show that moderate MS effectively mitigates the deleterious effects of CSDS in adulthood at both behavioral and electrophysiological levels. It alleviates the increased anxiety-like behavior induced by CSDS, weakens CSDS-induced enhancement of excitatory synaptic transmission onto BLA PNs and mitigates CSDS-evoked augmentation of intrinsic excitability in these cells.

The duration and severity of early life stress exposure is tightly associated with the long-lasting stress effects. For instance, prolonged maternal separation and social isolation in infant rhesus monkeys were shown to produce an increased stress response and despair-like behavior in adults [26]. The mild to moderate separation (10 weekly 1-h social separation from 17-week to 27-week old), however, led to emotionally stable responses under stressful situations in squirrel monkeys and resulted in more exploration of novel settings [11], as well as improved prefrontal dependent cognitive control [27] and enhanced curiosity indicated by novelty-seeking [28]. Consistently, we observed that moderate MS ameliorated the increased anxiety-like behavior induced by CSDS in adulthood. The distinct influences of stress with varying duration and severity is generally believed to be associated with the divergent hypothalamus-pituitary-adrenal (HPA) axis responsiveness. The prolonged and severe stress enhances hypothalamus-pituitary-adrenal (HPA) axis responsiveness with deleterious consequences [29, 30], while the mild to moderate stress results in lower responsiveness and has beneficial, salutary effects. For example, early life stress inoculation including brief maternal separations and neonatal corticosterone administration, resulted in blunted HPA axis response to stress in adulthood [31, 32]. By contrast, it has also been demonstrated that stress inoculation acutely activated the glucocorticoid signaling during the training sessions of stress inoculation [14]. The mechanisms underlying such distinct effects of stress inoculation are not fully understood.

Previous studies have demonstrated that multiple brain regions are involved in the process of stress inoculation including mPFC, ventral striatum and hippocampus. At the molecular level, Lyons DM demonstrated that stress inoculation upregulates the expression of PEMT, (a key gene involved in acetylcholine biosynthesis) and CACNG2 (a gene that encodes stargazin) in ACC, while decreases that of SPRY2, a gene that encodes a negative regulator of neurotrophic factor signaling [33]. In addition, stress inoculation increases glucocorticoid receptor (NR3C1) expression in ACC, which is not associated with significant changes in glucocorticoid receptor (GR1F) promoter DNA methylation [14]. Here, we observed that MS alleviated the changes of synaptic transmission and neural activity of BLA neurons induced by CSDS in adults, suggesting amygdala may also play an important role in mediating the effect of stress inoculation.

Several lines of evidence have shown that glutamatergic neurotransmission is involved in the pathophysiology of stress induced psychiatry disorders [34, 35]. For instance, chronic restraint stress increases the frequency of mEPSC in amygdala projection neurons, which is positively correlated with the increased anxiety-like behavior [34]. On the other hand, CSDS reduced glutamatergic neurotransmission by decreasing the amplitude but not frequency of mEPSC in hippocampal pyramidal neurons. Notably, no significant effect on the amplitude of mEPSC was observed in resilient mice, which maintained normal psychological and physical functioning and avoided depression- and anxiety-like behavior when exposed even to extreme levels of stress [36]. Additionally, it has been demonstrated that neither mEPSC frequency nor amplitude was altered in the dentate gyrus in resilient mice [37]. These findings imply that alleviation of stress-induced changes of mEPSC may mitigate anxiety-like behavior. Consistently, we found that stress inoculation, as a form of resilience, prevented CSDS-evoked augmentation of mEPSC frequency but not amplitude in amygdala principal neurons. However, resilience increased mEPSC frequency but not amplitude in mPFC [38]. One possible explanation for this discrepancy is that the influence of stress on glutamatergic transmission differs among various brain regions. Likewise, chronic stress reduces dendritic arborization and glutamatergic dendritic spine density of pyramidal neurons in PFC and hippocampus, but increases dendritic spine number and dendritic branching in amygdala [2].

It is widely accepted that the changes of presynaptic glutamate release, as indicated by paired pulse ratio (PPR), may underlie the altered mEPSC frequency. Thus, in the current study, the stress inoculation (moderate MS) may mitigate later life stress-induced anxiety-like behavior through reducing the presynaptic glutamate release. Accumulating evidence indicates that multiple presynaptic molecules such as GABA_B_ receptors, adenosine A1, metabotropic glutamate receptors (mGluR) [39] and cannabinoid CB1 receptor [40] could affect the frequency of mEPSC by modulating presynaptic glutamate release. Whether these presynaptic molecules are involved in mediating the effects of stress inoculation on glutamatergic neurotransmission in amygdala needs to be further explored. In addition, the dendritic spine density which is the postsynaptic component of glutamatergic signaling which is a critical locus for synaptic plasticity [36], also contributes to the alteration of mEPSC frequency [34]. One interesting avenue for future studies would be identifying the effect of stress inoculation by moderate MS on the spine density of BLA PNs.

Our results showed neither CSDS nor stress inoculation had any obvious effects on the frequency or amplitude of mIPSC in BLA PNs, suggesting stress inoculation may not modulate presynaptic GABA release or postsynaptic GABA_A_R expression. Consistent with our findings, our previous study showed that chronic stress does not affect phasic GABA_A_R currents (as indicated by sIPSC) [18]. Moreover, it has been recently demonstrated that resilience did not affect inhibitory synaptic transmission onto hippocampal dentate gyrus neurons [38] or NAc GABAergic neurons [41]. However, one study reported that the amplitude of mIPSC and eIPSC of amygdala neurons was increased in resilient mice [42]. One possible explanation for this discrepancy may lie in the difference of animal models (C57BL/6 J mice in the present study, Wistar rats in the previous study [42]) and resilience (achieved by stress inoculation in the present study, while NPY-induced resilience in the previous study). It is worth noting that chronic stress has been shown to reduce the tonic GABA_A_R currents that mediated by extrasynaptic GABA_A_Rs. Whether stress inoculation can attenuate stress-induced tonic current changes needs to be further investigated.

Overexcitation of principal neurons in BLA has been shown to be highly associated with stress-related behavior [23], while decreasing the excitability of amygdala neurons by NPY induces resilience [43]. We here observed that stress inoculation attenuated CSDS-induced increase of intrinsic excitability in BLA PNs in the present study. Of note, several cellular characteristics contributing to the neuronal intrinsic excitability including resting membrane potential, threshold, amplitude and rheobase of action potential remained unaltered by CSDS or stress inoculation. Moreover, accumulating evidence has demonstrated that reduced afterhyperpolarization (AHP) amplitude in BLA PNs is closely associated with increased intrinsic excitability [23, 44], while our present study showed that stress inoculation had little effect on the fast AHP (fAHP). However, since medium (mAHP) and slow AHPs (sAHP) have also been reported to play essential role in regulating action potential [23], whether mAHP and sAHP contribute to the alteration of intrinsic excitability by stress inoculation remains to be determined.

In summary, our results indicated that stress inoculation could mitigate CSDS-induced anxiety-like behavior by alleviating CSDS-induced enhancement of glutamate transmission and intrinsic excitability. Despite this, several important questions remain open. For example, although we demonstrated the synaptic and cellular mechanisms of stress inoculation in amygdala, the underlying molecular mechanism remains elusive. On the other hand, the neurons are extensively intermingled within amygdala; however, they differ drastically in terms of their connectivity and functionality [45]. Our recent studies demonstrated that chronic stress specifically regulated the structural and functional changes of vHPC projecting (BLA → vHPC) but not dmPFC projecting (BLA → dmPFC) or NAc projecting (BLA → NAc) neurons in BLA [23, 34]. Thus, whether stress inoculation may have distinct effect on these BLA neurons projecting to different brain regions (such as mPFC, NAc, vHPC, BNST) remains unclear at the synaptic and cellular levels. Addressing these issues will help to understand the molecular and circuit mechanisms of stress inoculation in amygdala, and may provide a more precise intervention approach for the prevention of stress-related disorders.

## Supplementary information

**Additional file 1: Figure S1.** No significant effect on the total locomotion and closed arm entries in the EPM, and a time course of locomotion in the OF tests by MS and CSDS. **a** Comparison of the total distance travelled in the elevated plus maze (Control: *n* = 15 mice, CSDS: *n* = 13 mice, MS: *n* = 16 mice, MS + CSDS: *n* = 16 mice). **b** Comparison of entries in closed arms in the elevated plus maze (Control: *n* = 15 mice, CSDS: *n* = 13 mice, MS, MS + CSDS: *n* = 16 mice). **c** Comparison of time course of locomotion in the open field test (Control: *n* = 12 mice, CSDS: *n* = 14 mice, MS, MS + CSDS: *n* = 11 mice). All data are presented as the mean ± SEM.

**Additional file 2: Figure S2.** Mitigation of CSDS-induced enhancement of intrinsic excitability by MS independent of synaptic input in BLA PNs. **a** Representative spiking traces showing the firing of BLA PNs upon the injection of depolarizing current with the ramped strength (0-250pA, 1500 ms) in the presence of glutamate receptor antagonist (20 μM CNQX and 25 μM APV) and GABA_A_ receptor antagonist (100 μM picrotoxin) to block synaptic transmission. **b** Comparison of the firing frequency (numbers of APs). *p < 0.05. All data are presented as the mean ± SEM.

**Additional file 3: Figure S3.** No significant effect on AP threshold, rheobase, AP amplitude and fAHP by MS and CSDS. **a** Effect of MS on the threshold of action potential in BLA PNs. **b** Effect of MS on rheobase in BLA PNs. **c** Effect of MS on the amplitude of action potential in BLA PNs. **d** Effect of MS on fAHP in BLA PNs. All data are presented as the mean ± SEM.

## Data Availability

Not applicable.

## Abbreviations

BNST: Bed nucleus of the stria terminalis
CSDS: Chronic social defeat stress
mEPSC: Miniature excitatory post synaptic current
mIPSC: Miniature inhibitory post synaptic current
mPFC: Medial prefrontal cortex
MS: Maternal separation
NAc: Nucleus accumbens
PNs: Principal neurons
vHPC: Ventral hippocampus
