# Supplementary figures and images for: Moderate maternal separation mitigates the altered synaptic transmission and neuronal activation in amygdala by chronic stress in adult mice
Source: Mol Brain. 2019 Dec 18;12:111. doi: 10.1186/s13041-019-0534-4 (PMC6918580; doi:10.1186/s13041-019-0534-4)

# Figure S1

a

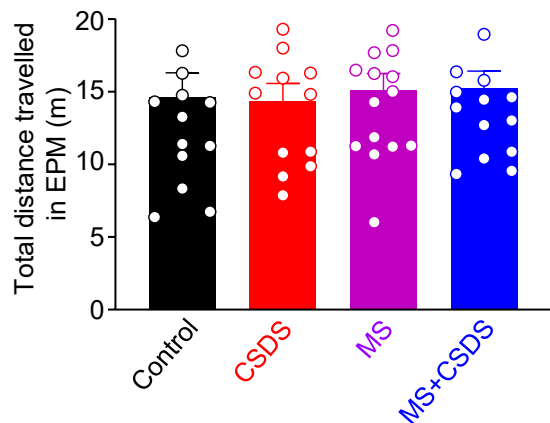

b

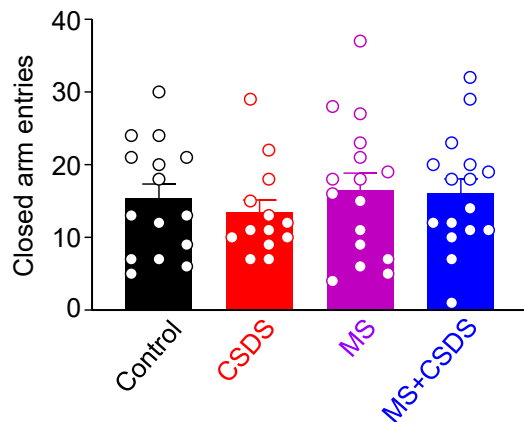

c

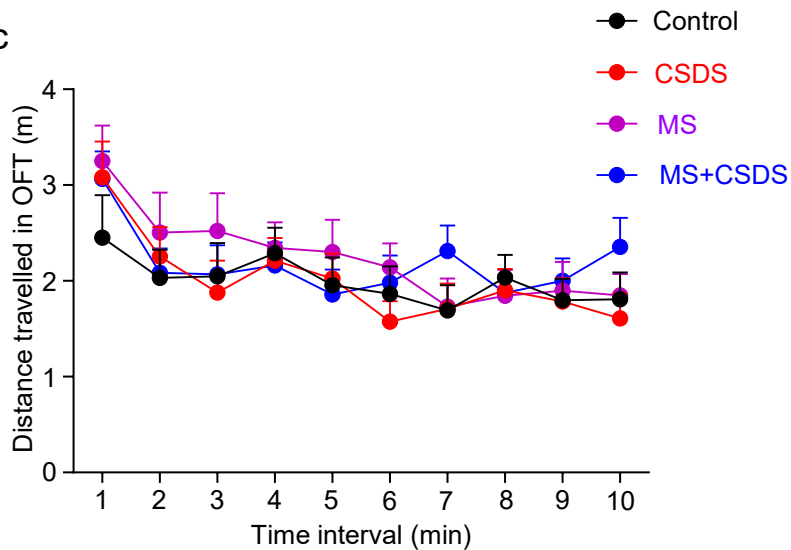

Supplement: Supplementary file 1 — Additional file 1: Figure S1. No significant effect on the total locomotion and closed arm entries in the EPM, and a time course of locomotion in the OF tests by MS and CSDS. a Comparison of the total distance travelled in the elevated plus maze (Control: n = 15 mice, CSDS: n = 13 mice, MS: n = 16 mice, MS + CSDS: n = 16 mice). b Comparison of entries in closed arms in the elevated plus maze (Control: n = 15 mice, CSDS: n = 13 mice, MS, MS + CSDS: n = 16 mice). c Comparison of time course of locomotion in the open field test (Control: n = 12 mice, CSDS: n = 14 mice, MS, MS + CSDS: n = 11 mice). All data are presented as the mean ± SEM. [file 13041_2019_534_MOESM1_ESM.pdf]

Figure S2

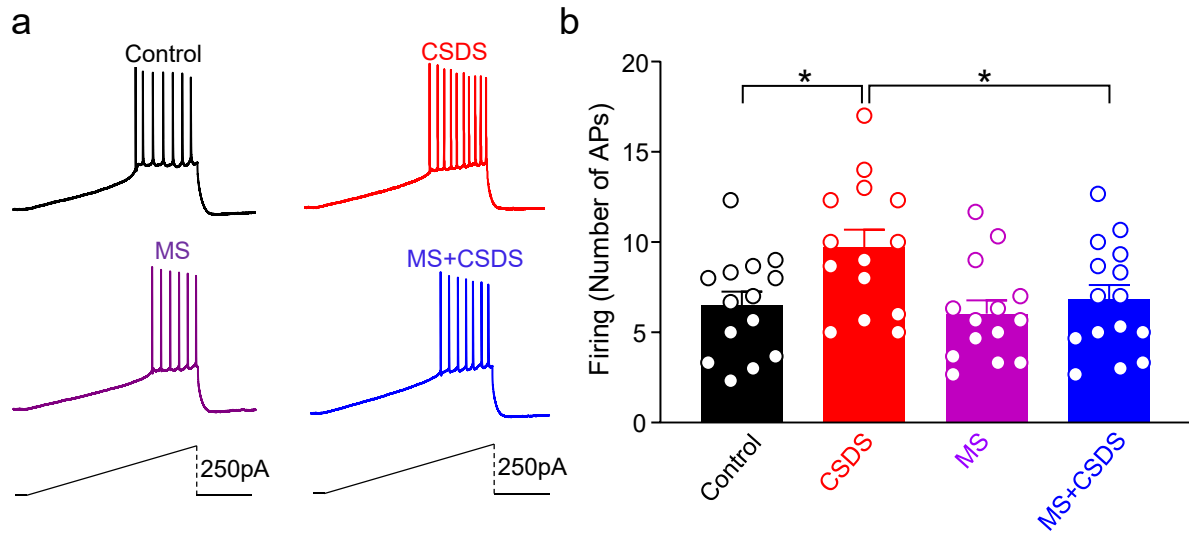

Supplement: Supplementary file 2 — Additional file 2: Figure S2. Mitigation of CSDS-induced enhancement of intrinsic excitability by MS independent of synaptic input in BLA PNs. a Representative spiking traces showing the firing of BLA PNs upon the injection of depolarizing current with the ramped strength (0-250pA, 1500 ms) in the presence of glutamate receptor antagonist (20 μM CNQX and 25 μM APV) and GABAA receptor antagonist (100 μM picrotoxin) to block synaptic transmission. b Comparison of the firing frequency (numbers of APs). *p < 0.05. All data are presented as the mean ± SEM. [file 13041_2019_534_MOESM2_ESM.pdf]
