# Supplementary material for: Moderate maternal separation mitigates the altered synaptic transmission and neuronal activation in amygdala by chronic stress in adult mice
Source: Mol Brain. 2019 Dec 18;12:111. doi: 10.1186/s13041-019-0534-4 (PMC6918580; doi:10.1186/s13041-019-0534-4)
Supplement: Supplementary file 3 — Additional file 3: Figure S3. No significant effect on AP threshold, rheobase, AP amplitude and fAHP by MS and CSDS. a Effect of MS on the threshold of action potential in BLA PNs. b Effect of MS on rheobase in BLA PNs. c Effect of MS on the amplitude of action potential in BLA PNs. d Effect of MS on fAHP in BLA PNs. All data are presented as the mean ± SEM. [file 13041_2019_534_MOESM3_ESM.pdf]

Figure S3

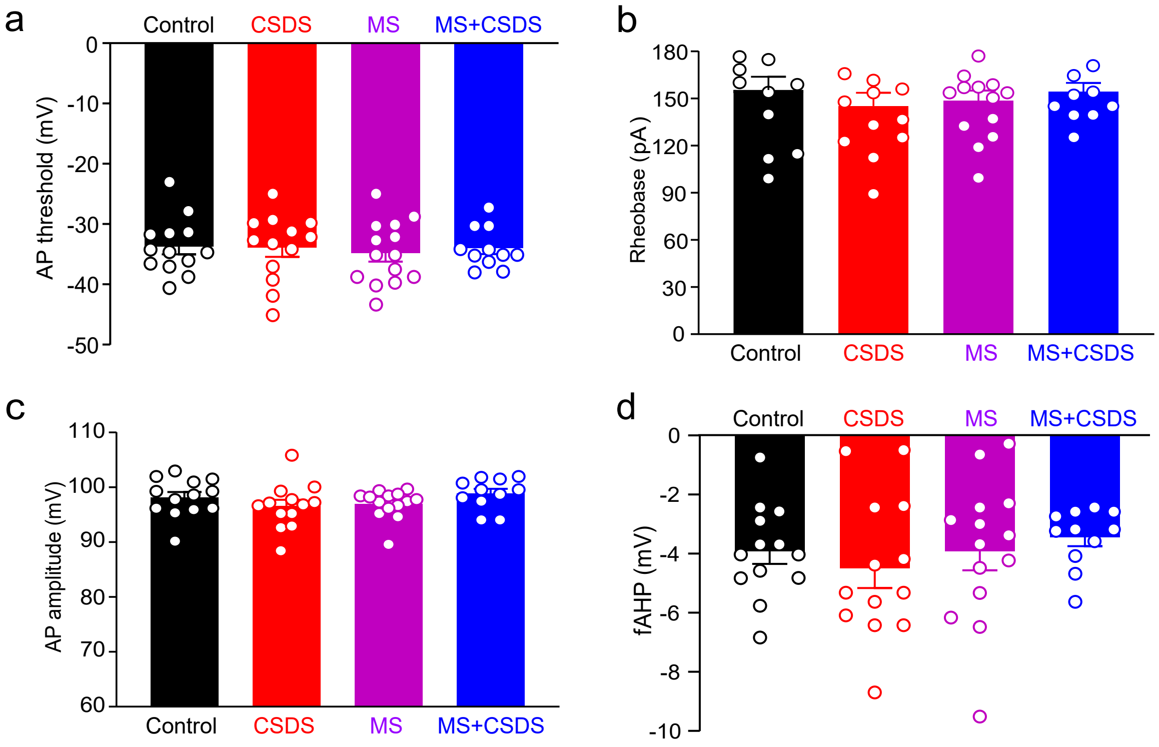
a
Control
CSDS
MS
MS+CSDS
AP threshold (mV)
0
-10
-20
-30
-40
-50
b
Rheobase (pA)
180
150
120
90
60
30
0
Control
CSDS
MS
MS+CSDS
c
AP amplitude (mV)
110
100
90
80
70
60
Control
CSDS
MS
MS+CSDS
d
Control
CSDS
MS
MS+CSDS
fAHP (mV)
0
-2
-4
-6
-8
-10
